# Dimethyl fumarate induces a persistent change in the composition of the innate and adaptive immune system in multiple sclerosis patients

**DOI:** 10.1038/s41598-018-26519-w

**Published:** 2018-05-29

**Authors:** G. Montes Diaz, J. Fraussen, B. Van Wijmeersch, R. Hupperts, V. Somers

**Affiliations:** 1 Hasselt University, Biomedical Research Institute and Transnationale Universiteit Limburg, School of Life Sciences, Diepenbeek, Belgium; 2 Rehabilitation & MS Center, Overpelt, Belgium; 3 Department of Neuroscience, School of Mental Health and Neuroscience, Maastricht University, Maastricht, The Netherlands; 4 Department of Neurology, Academic MS Center Limburg, Zuyderland Medisch Centrum, Sittard, The Netherlands

## Abstract

The effects of dimethyl fumarate (DMF) on the immune system in multiple sclerosis (MS) are not completely elucidated. In this study, an extensive immunophenotypic analysis of innate and adaptive immune cells of DMF-treated MS patients was performed. Peripheral blood immune cell phenotypes were determined using flow cytometry in a follow-up study of 12 MS patients before, after 3 and 12 months of DMF treatment and a cross-sectional study of 25 untreated and 64 DMF-treated MS patients. Direct effects of DMF on B cells were analyzed *in vitro*. After 12 months of DMF treatment, percentages of monocytes, natural killer cells, naive T and B cells and transitional B cells increased. Percentages of (effector) memory T cells, (non) class-switched memory B cells and double negative B cells decreased together with CD4^+^ T cells expressing interferon-γ (IFN-γ), granulocyte macrophage colony-stimulating factor (GM-CSF) and interleukin-17 (IL-17). DMF treatment was fully effective as of 6 months and directly induced apoptosis and decreased expression of costimulatory CD40, antigen presentation molecule MHCII and B cell activating factor receptor (BAFFR) on B cells. DMF induced a persistent change of the immune system of MS patients, directly induced apoptosis and reduced expression of functional markers on B cells.

## Introduction

MS is a chronic inflammatory disease of the CNS in which both the innate and adaptive immune system are involved. The immune system of MS patients displays a disrupted balance favoring pro-inflammatory responses. T cells have long been considered as the most important players although the emergence of B cell depleting treatment has emphasized the importance of B cells in MS pathogenesis^1,2^. Adaptive immune cell subtypes involved in the pathological processes during MS are memory T cells (CD45RO^+^CD45RA^−^) and more specifically C-C chemokine receptor type 7 (CCR7)^−^ effector memory T cells, CD8^+^ T cells and memory (CD27^+^) B cells^3–8^. CD4^+^ T helper (Th) 1 and Th17 cells produce pro-inflammatory cytokines including interferon-γ (IFN-γ), interleukin-17 (IL-17) and granulocyte macrophage colony-stimulating factor (GM-CSF) while Th2 and regulatory T (Treg) cells produce anti-inflammatory IL-4 and IL-10^1,9^. Double negative (DN, IgD^−^CD27^−^) B cells with a pro-inflammatory cytokine profile are described to be increased in a proportion of MS patients^10^. For the innate immune system, monocytes (CD14^+^), neutrophils and natural killer (NK) cells, including CD56^dim^ and CD56^bright^ NK cells, are involved in MS pathogenesis^11–14^.

Dimethyl fumarate (DMF, Tecfidera^®^) was licensed as an oral first-line treatment for MS in 2013. Two phase III clinical trials, DEFINE and CONFIRM, demonstrated clinical efficacy of DMF in relapsing-remitting (RR) MS^15,16^. DMF reduced the annualized relapse rate and reduced the mean number of new or enlarging MRI lesions throughout the course of the study^15,16^. Earlier studies have demonstrated that DMF and its active metabolite monomethyl fumarate (MMF) exert neuroprotective and immunomodulatory effects through the activation of transcription factor nuclear factor (erythroid-derived 2)-like 2 (Nrf2) and via the suppression of transcription factor nuclear factor kappa b (NF-kB)^17,18^.

A number of studies investigated the effect of DMF on certain subtypes of disease-related immune cells^19,20^. Nevertheless, the working mechanism of DMF is multifactorial and the pathogenesis of MS is directed by interplay between different immune cell subtypes. Previous studies were limited by a cross-sectional design, limited follow-up to 6 months only, a focus on 1 immune cell subtype or the inclusion of patients who were previously treated with second-line therapies. Therefore, we performed an extensive immunophenotypic analysis of the innate and adaptive immune system of RRMS patients under DMF treatment using 3 study designs: a 12 month (m) follow-up study, a cross-sectional study and an *in vitro* study. In this way we further explored changes of several immune cell types over a longer period in a cause-and-effect way in untreated MS patients or in MS patients previously treated with first-line treatments.

## Results

### Clinical characteristics

In the follow-up study, 16 RRMS patients were enrolled and peripheral blood was collected before treatment, after 3 m and after 12 m of DMF treatment. Twelve of the 16 MS patients finished the total duration of the study (Table 1), while 4 MS patients dropped out of the study, with 2 due to side effects (gastrointestinal and flushing), 1 due to pregnancy and 1 due to other medication use. Eight of the 12 DMF treated MS patients underwent MRI before and after 12 m of DMF treatment. In all patients, no new or enlarged lesions were detected. Furthermore, 4 of these 8 MS patients showed lesions that were decreased in volume or were less pronounced compared to baseline. Although not significant, EDSS decreased from 2.8 at baseline to 2.3 after 12 m of DMF treatment (p = 0.0547, Table 2). When considering individual MS patients, EDSS improved for 6 patients, remained stable for 4 patients and increased for 2 patients who were clinical responders. Interestingly, a significantly improved cognitive function measured by the PASAT was observed after 3 m of DMF treatment (p < 0.05). Other clinical measures remained stable over the course of the study (Table 2).

**Table 1:** Characteristics of study subjects.

Group	N	Age^a^	Female % (F/M)^b^	EDSS^c^	Previous treatment
**Follow-up study**					
RRMS DMF-treated	12	34 ± 12	58 (7/5)	2.8 ± 1.2	UT: 8, IFN: 4
**Cross-sectional study**					
HC	10	30 ± 6	60 (6/4)	N.A.	N.A.
RRMS UT	25	36 ± 11	72 (18/7)	2.5 ± 1	UT: 19, IFN: 4, GA: 2
RRMS DMF-treated 0–5 m	23	37 ± 9	61 (14/9)	2.1 ± 1.2	UT: 15, IFN: 5, TF: 3
RRMS DMF-treated 6–12 m	23	36 ± 10	70 (16/7)	1.7 ± 1.2	UT: 10, IFN: 10, GA: 1, TF: 1
RRMS DMF-treated > 12 m	18	35 ± 10	72 (13/5)	1.6 ± 1.4	UT: 3, IFN: 12, GA: 2, TF: 1
***In vitro*** **study**					
Costimulatory molecules					
HC	9	37 ± 12	55 (5/4)	N.A.	N.A.
RRMS UT	7	45 ± 11	57 (4/3)	2.8 ± 1.8	UT: 6, IFN: 1
Apoptosis					
HC	12	34 ± 11	67 (8/4)	N.A.	N.A.
RRMS UT	6	45 ± 8	66 (4/2)	2.8 ± 1.9	UT: 4, IFN: 2
BAFFR					
HC	14	33 ± 11	71 (10/4)	N.A	N.A.
RRMS UT	6	45 ± 8	66 (4/2)	2.8 ± 1.9	UT: 4, IFN: 2
Breg frequency					
RRMS UT	5	38 ± 13	80 (4/1)	2 ± 0.8	UT: 5

^a^Mean age in years; ^b^number of female (F) and male (M) individuals; ^c^mean Expanded Disability Status Scale (EDSS); IFN = interferon-β, RRMS = relapsing-remitting multiple sclerosis, DMF = dimethyl fumarate, HC = healthy control, m = months, Breg = regulatory B cell, N.A. = not applicable, UT = untreated, TF = teriflunomide, GA = glatiramer acetate.

**Table 2:** Clinical tests and absolute numbers of immune cells after DMF treatment.

Clinical test	Baseline mean ± SD	3 m DMF mean ± SD	12 m DMF mean ± SD
EDSS	2.8 ± 1.3	2.7 ± 1.6	2.3 ± 1.7^§^
PASAT	50 ± 4	53 ± 3	53 ± 4^*^
NHPT dominant hand	20.6 s ± 5	20 s ± 4	19 s ± 3
NHPT non-dominant hand	20.3 s ± 4	20 s ± 3	19.8 s ± 4
T25FW	4.9 s ± 2	5.5 s ± 3	5.5 s ± 3
FSMC (motor)	31 ± 7	31 ± 7	31 ± 6
FSMC (cognitive)	30 ± 9	31 ± 10	32 ± 11
**Absolute number (x10** ^**3**^ **/ml)** ^**a**^ **(clinic)**			
Leukocytes	7984 ± 1862	7180 ± 2905	6397 ± 1754^*^
Lymphocytes	2494 ± 746	2019 ± 809	1346 ± 289^*^
Monocytes	488 ± 137	501 ± 96	453 ± 84
Neutrophils	4826 ± 1302	4514 ± 1962	4470 ± 1616
**Absolute number (x10** ^**3**^ **/ml)** ^**a**^ **(calculated)**			
CD56^dim^ NK cells	160 ± 77	156 ± 112	111 ± 53^*^
CD56^+^ NK cells	16 ± 9	20 ± 14	19 ± 8^*^
T cells (CD3^+^)	1857 ± 669	1520 ± 604	974 ± 270^*^
CD4^+^ T cells	1072 ± 268	955 ± 379	623 ± 197^*^
Naive CD4^+^ T cells (CD4^+^CD45RO^−^CD45RA^+^)	439 ± 246	453 ± 226	340 ± 195
Memory CD4^+^ T cells (CD4^+^CD45RO^+^CD45RA^−^)	623 ± 156	523 ± 295	279 ± 153^*^
Regulatory T cells (CD4^+^CD25^+^CD127^low^)	53 ± 14	44 ± 20	27 ± 10^*^
IL-17^+^ CD4^+^ T cells	6 ± 4	4 ± 3	3 ± 2
GM-CSF^+^ CD4^+^ T cells	65 ± 33	49 ± 46	19 ± 14^*^
IFN-γ^+^ CD4^+^ T cells	99 ± 33	66 ± 43	31 ± 14^*^
IL-4^+^ CD4^+^ T cells	34 ± 11	25 ± 14	18 ± 10^*^
IL-10^+^ CD4^+^ T cells	3 ± 3	5 ± 5	2 ± 3^*^
CD8^+^ T cells	618 ± 452	447 ± 261	254 ± 117^*^
Naive CD8^+^ T cells (CD8^+^CD45RO^−^CD45RA^+^)	402 ± 366	281 ± 191	211 ± 104
Memory CD8^+^ T cells (CD8^+^CD45RO^+^CD45RA^−^)	210 ± 180	164 ± 176	42 ± 23^*^
B cells (CD19^+^)	295 ± 165	187 ± 132	142 ± 47^*^
Transitional B cells (CD19^+^CD27^−^CD38^+^)	8 ± 5	8 ± 4	7 ± 4
Naive B cells (CD19^+^CD27^−^IgD^+^)	185 ± 140	120 ± 90	102 ± 46
Non class-switched memory B cells (CD19^+^CD27^+^IgD^+^)	38 ± 24	18 ± 8	17 ± 8^*^
Class-switched memory B cells (CD19^+^CD27^+^IgD^−^)	41 ± 13	24 ± 16	15 ± 4^*^
Double negative B cells (CD19^+^CD27^−^IgD^−^)	12 ± 5	8 ± 4	6 ± 2^*^

DMF = dimethyl fumarate, m = months, ^§^p = 0.05, ^*^p < 0.05 12 m compared to baseline.

^a^Absolute numbers of immune cells were available from 7 of the 12 DMF-treated MS patients, EDSS was available from all DMF-treated MS patients while FSMC and NHPT was available from 9/12 DMF-treated MS patients and PASAT and T25FW was available from 8/12 DMF-treated MS patients.

### Absolute numbers of immune cells

Absolute immune cell numbers of leukocytes, lymphocytes, monocytes and neutrophils were obtained from standard clinical lab tests for 7 DMF-treated MS patients. A significant decrease in the absolute leukocyte (20%) and lymphocyte (46%) numbers was measured after 12 m of DMF treatment (p = 0.0313, Table 2). Grade 3 lymphopenia (absolute lymphocyte numbers <0.5 × 10^9^ cells/L) was not observed in this patient cohort. Absolute monocyte and neutrophil numbers remained stable. Specific cell subtypes of both the innate and adaptive immune system, calculated as described in the methods section, showed significantly reduced numbers after 12 m but not after 3 m of DMF treatment. The absolute number of CD56^dim^ NK cells was significantly reduced (p = 0.0156), while CD56^bright^ NK cell numbers were significantly increased (p = 0.0469). The absolute numbers of T cells (CD3^+^) and all T cell subtypes were significantly decreased (p < 0.05), except for naive T cells (p = 0.1563 for CD4^+^CD45RO^−^CD45RA^+^ and p = 0.0781 for CD8^+^CD45RO^−^CD45RA^+^) and IL-17^+^ CD4^+^ T cells (p = 0.1094). Within the B cell population, the absolute numbers of total B cells (CD19^+^) and all B cell subtypes were decreased (p < 0.05) except for transitional B cells (p = 0.6875) and naive B cells (p = 0.0781), which were not affected. In conclusion, most immune cell subtypes showed decreased circulating absolute numbers following 12 m of DMF treatment. Therefore, it was important to investigate the remaining peripheral blood immune cell population by determining immune cell subtype percentages.

### Percentages of innate immune cell subtypes

**Figure 1:**
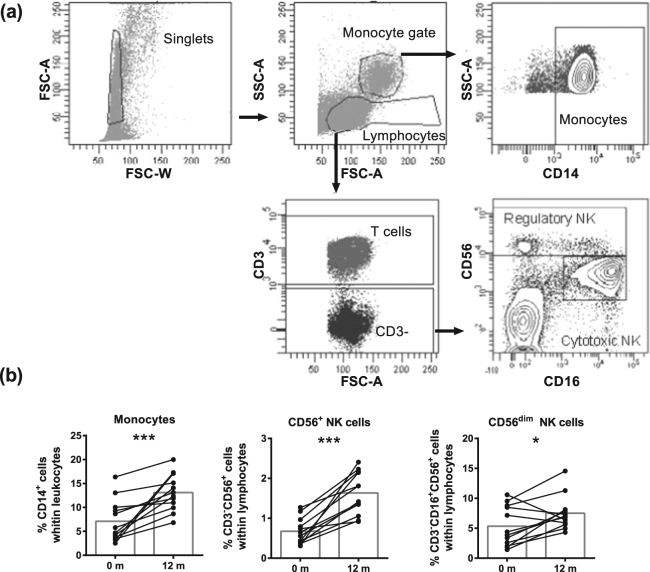
DMF treatment increased frequencies of monocytes and NK cells. (**a**) A representative analysis of innate immune cell subtypes is shown. Single cells were selected using forward scatter (FSC) area and width. Lymphocytes were gated in the singlet gate using FSC and side scatter (SSC) parameters. CD56^dim^ NK cells (CD3^−^CD16^+^CD56^dim^) and CD56^+^ NK cells (CD3^−^CD16^+^CD56^+^) were gated from lymphocytes and the CD3- cell population. Monocytes (CD14^+^) were gated from monocyte gate. (**b**) Frequency of monocytes and NK cells at baseline and after 12 m of DMF treatment in RRMS patients (n = 12). Each line represents an individual patient and average values are depicted as histograms. Wilcoxon matched-pairs signed rank test was used to determine p values. ^*^p < 0.05, ^**^p < 0.01, ^***^p < 0.001. NK = natural killer, m = months.

When analyzing innate immune cell subtypes (gated as in Fig. 1a), increased percentages of monocytes (p = 0.0005), CD56^+^ (p = 0.0005) and CD56^dim^ (p = 0.0269) NK cells were detected after 12 m (Fig. 1b), but not after 3 m (Supplementary Fig. 1) of DMF treatment. Thus, DMF treatment increased the percentages of all analyzed innate immune cell subtypes.

### Percentages of adaptive immune cell subtypes

**Figure 2:**
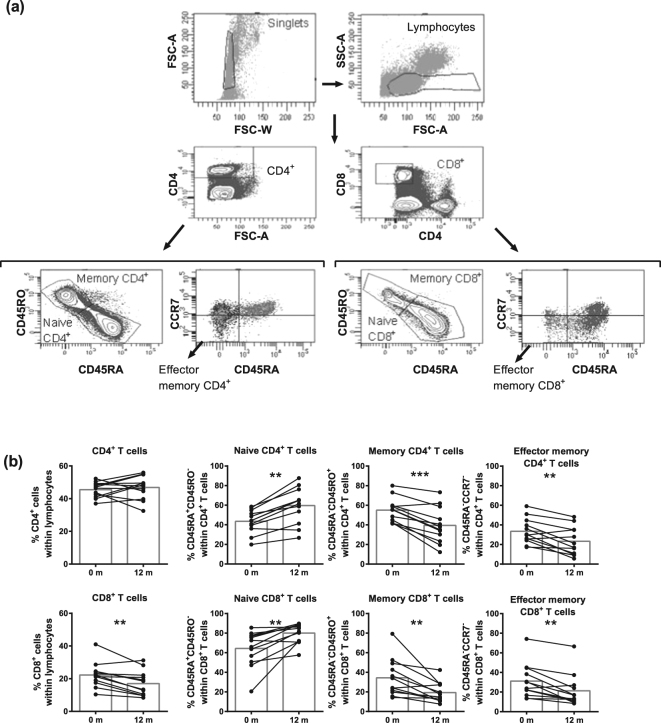
DMF treatment reduced (effector) memory T cell frequencies after 12 m of treatment in MS patients. (**a**) A representative analysis of T cell subtypes is shown. Single cells were selected using FSC area and width. Lymphocytes were gated within the singlet gate using FSC and SSC parameters. Next, CD4^+^ T cells and CD8^+^ T cells were gated from lymphocytes. Naive T cells (CD45RA^+^CD45RO^−^), memory T cells (CD45RA^−^CD45RO^+^) and effector memory T cells (CD45RA^−^CCR7^−^) were gated from the CD4^+^ or CD8^+^ T cell population. (**b**) Frequency of T cell subtypes at baseline and after 12 m of DMF treatment in RRMS patients (n = 12). Each line represents an individual patient and average values are depicted as histograms. Wilcoxon matched-pairs signed rank test was used to determine p values. ^*^p < 0.05, ^**^p < 0.01, ^***^p < 0.001. m = months.

**Figure 3:**
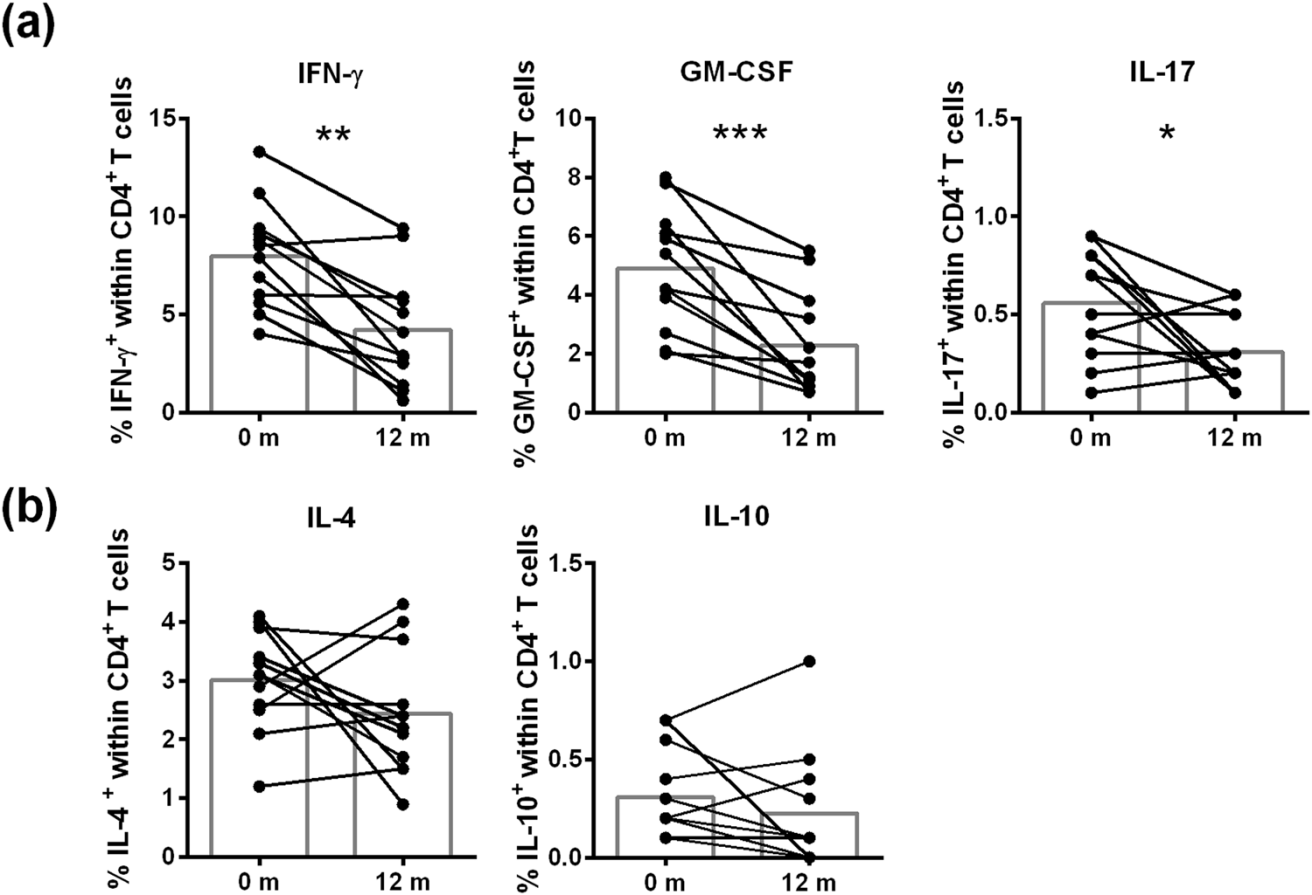
Reduction in the frequency of T cells expressing pro-inflammatory cytokines after 12 m of DMF treatment. Frequency of CD4^+^ T cells expressing pro-inflammatory cytokines (**a**), including IFN-γ, GM-CSF, IL-17 or anti-inflammatory cytokines (**b**), including IL-4 and IL-10, in RRMS patients at baseline and after 12 m of DMF treatment (n = 12). Each line represents an individual patient and average values are depicted as histograms. Wilcoxon matched-pairs signed rank test was used to determine p values. ^*^p < 0.05, ^**^p < 0.01, ^***^p < 0.001. IFN-γ = interferon γ, GM-CSF = granulocyte macrophage colony-stimulating factor, IL-17 = interleukin-17, IL-4 = interleukin-4, IL-10 = interleukin-10, m = months.

After 12 m of DMF treatment, T cell percentages decreased, although not significantly (data not shown). Within the T cell population (gated as in Fig. 2a), CD4^+^ T cells remained stable, while CD8^+^ T cells decreased (p = 0.0034, Fig. 2b). Most importantly, a decrease in the percentages of memory and effector memory CD4^+^ and CD8^+^ T cells was evident (p < 0.01) while naive CD4^+^ and CD8^+^ T cell percentages increased (p < 0.01). No effect of DMF treatment was observed on Treg percentages (Supplementary Fig. 1). When analyzing cytokine-expressing CD4^+^ T cells (gating shown in Supplementary Fig. 2), decreased percentages of pro-inflammatory IFN-γ^+^ (p = 0.0015), GM-CSF^+^ (p = 0.0005) and IL-17^+^ (p = 0.0488) CD4^+^ T cells (Fig. 3a) were detected and anti-inflammatory IL-4^+^ and IL-10^+^ CD4^+^ T cells were unchanged (Fig. 3b, Supplementary Fig. 2).

**Figure 4:**
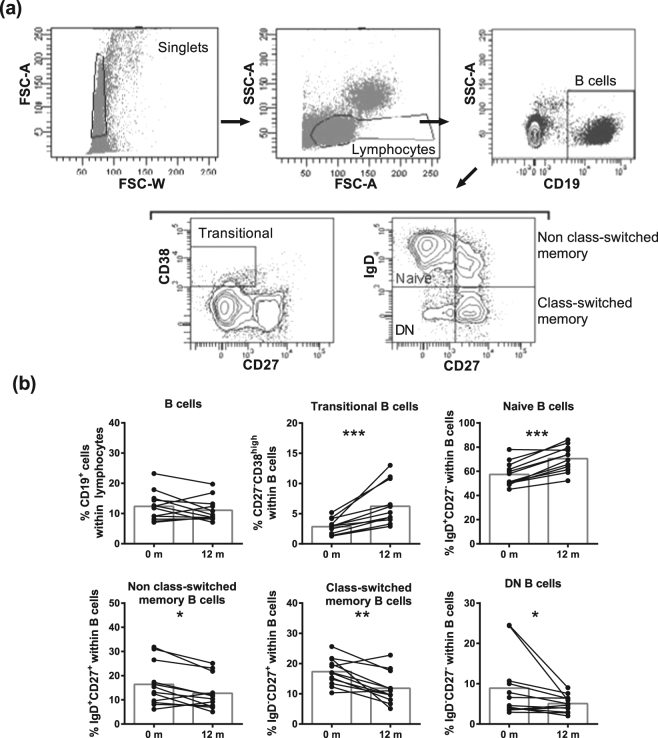
DMF treatment reduced memory and DN B cell subtypes after 12 m of treatment in MS patients. (**a**) A representative analysis of B cell subtypes is shown. Single cells were selected using FSC area and width. Lymphocytes were gated in the singlet gate using FSC and SSC parameters. B cells (CD19^+^) were gated from lymphocytes followed by analysis of transitional B cells (CD27^−^CD38^high^), naive B cells (CD27^−^IgD^+^), non class-switched memory B cells (CD27^+^IgD^+^), class-switched memory B cells (CD27^+^IgD^−^) and DN B cells (CD27^−^IgD^−^). (**b**) B cell subtypes at baseline and after 12 m of DMF treatment in RRMS patients (n = 12). Each line represents an individual patient and average values are depicted as histograms. Wilcoxon matched-pairs signed rank test was used to determine p values. ^*^p < 0.05, ^**^p < 0.01, ^***^p < 0.001. DN = double negative, m = months.

Furthermore, B cell percentages were not affected by DMF treatment (Fig. 4). Within the B cell population (gated as in Fig. 4a), percentages of transitional and naive B cells increased (Fig. 4b). Further, DMF treatment resulted in decreased percentages of DN (p = 0.0161), class-switched memory (p = 0.0034) and non class-switched memory (p = 0.0161) B cells. After 3 m of DMF treatment, no significant differences were observed in the percentages of all adaptive immune cell subtypes (Supplementary Fig. 1).

*In vitro* treatment of B cells from 5 untreated RRMS patients with DMF or MMF indicated that DMF induced a trend towards an increased regulatory B cell (Breg) percentage (p = 0.06, Supplementary Fig. 3). MMF decreased the percentage of TNF-α^+^ B cells, although not significantly (p = 0.06). Together, these results indicate that 12 m DMF treatment reduced percentages of pro-inflammatory and memory T and B cell subtypes and increased percentages of naive T and B cells and transitional B cells.

### T cell subtypes in a cross-sectional study

**Figure 5:**
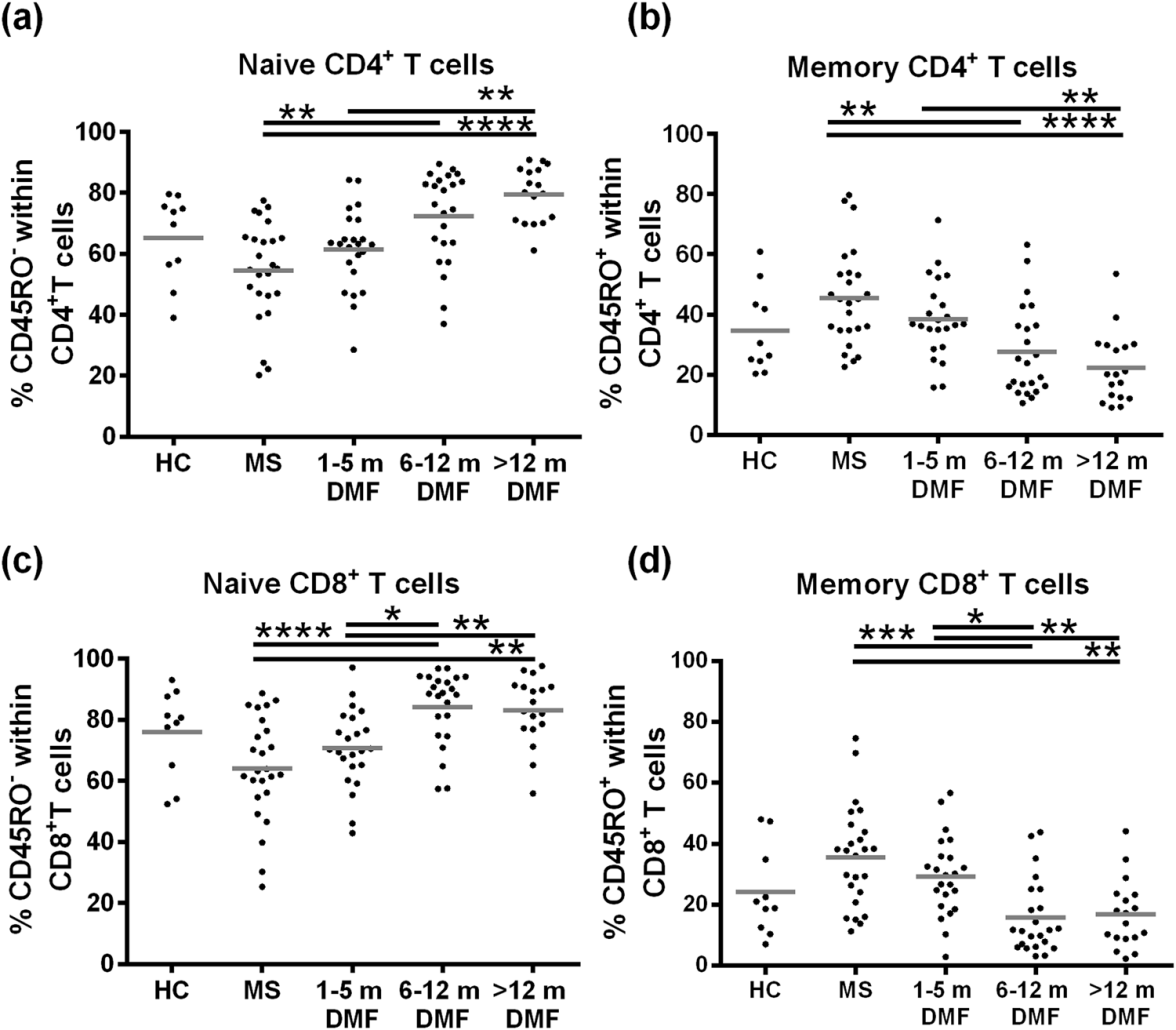
DMF treatment is fully effective on immune cells after 6 m of treatment. Frequencies of naive and memory CD4^+^ and CD8^+^ T cells in HC (n = 10), untreated RRMS patients (n = 25), 1–5 m DMF-treated RRMS patients (n = 23), 6–12 m DMF-treated RRMS patients (n = 23), >12 m DMF-treated MS patients (n = 18). A Kruskal-Wallis one-way ANOVA was used to compare the different groups. ^*^p < 0.05, ^**^p < 0.01, ^***^p < 0.001, ^****^p < 0.0001. HC = healthy control, DMF = dimethyl fumarate, m = months.

Since 3 m DMF treatment only partly reflected changes reported at 12 m, additional time points were included in a cross-sectional study to identify how soon the reported effect was found after treatment (Table 1). Memory CD4^+^ and CD8^+^ T cell percentages were reduced, while naive CD4^+^ and CD8^+^ T cell percentages were increased after 6 m of DMF treatment compared to untreated MS patients (Fig. 5). Furthermore, percentages of memory CD8^+^ T cells were decreased, while naive CD8^+^ T cells were increased after 6–12 m compared with 1–5 m of DMF treatment. After prolonged treatment (>12 m), memory and naive CD4^+^ and CD8^+^ T cell percentages remained stable. Thus, DMF is fully effective after 6 m of treatment.

### Direct effect of DMF on B cell apoptosis

**Figure 6:**
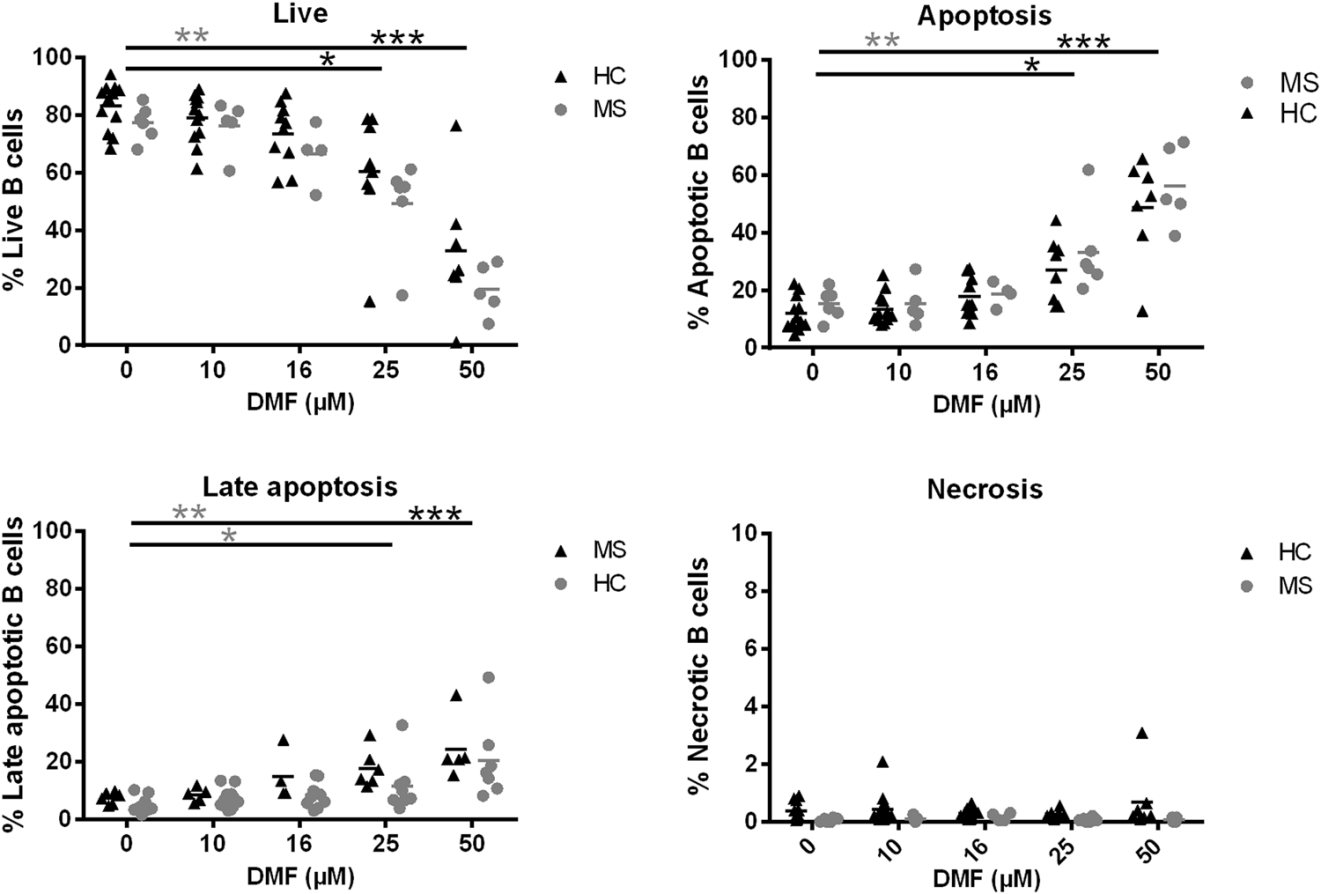
DMF induces apoptosis in B cells in a concentration-dependent manner. Purified B cells of HC and untreated RRMS patients were treated *in vitro* with the following concentrations of DMF: 10 µM (HC: n = 12, MS: n = 5), 16 µM (HC: n = 10, MS: n = 4), 25 µM (HC: n = 8, MS: n = 6) and 50 µM (HC: n = 7, MS: n = 5) or left untreated (HC: n = 12, MS: n = 6). (**a**) Frequency of live B cells (Annexin V^−^7-AAD^−^), (**b**) apoptotic B cells (Annexin V^+^7-AAD^−^), (**c**) late apoptotic B cells (Annexin V^+^7-AAD^+^) and (**d**) necrotic B cells (Annexin V^−^7-AAD^+^) of HC and MS patients. A Kruskal-Wallis one-way ANOVA was used to compare the different groups. ^*^p < 0.05, ^**^p < 0.01, ^***^p < 0.001. HC = healthy control, MS = multiple sclerosis, DMF = dimethyl fumarate.

We next investigated induction of apoptosis as one of the underlying mechanisms of the drop in absolute lymphocyte numbers. Previously, *in vitro* studies showed that DMF induced T cell apoptosis with a preferential effect on memory T cells^21^. Since DMF treatment decreased the percentage of memory B cells while increasing naive B cells, we investigated whether DMF induced apoptosis of B cells and whether naive B cells showed a lower vulnerability to DMF-induced apoptosis. Here, the direct effect of DMF and MMF on B cell apoptosis was investigated (Table 1). In HC, DMF induced B cell apoptosis at 25 µM (p < 0.05) and 50 µM (p < 0.001) compared to baseline (Fig. 6). In untreated MS patients, apoptosis was induced with 50 µM DMF (p < 0.01), although late B cell apoptosis was already induced at 25 µM (p < 0.05). In HC, late apoptosis was only induced at 50 µM DMF (p < 0.001). MMF treatment did not induce B cell apoptosis (Supplementary Fig. 4) and no difference was detected between memory and naive B cells (data not shown). In summary, DMF induced concentration-dependent apoptosis of B cells from HC and MS patients with B cells of MS patients appearing to be more vulnerable.

### Direct effect of DMF on functional markers of B cells

**Figure 7:**
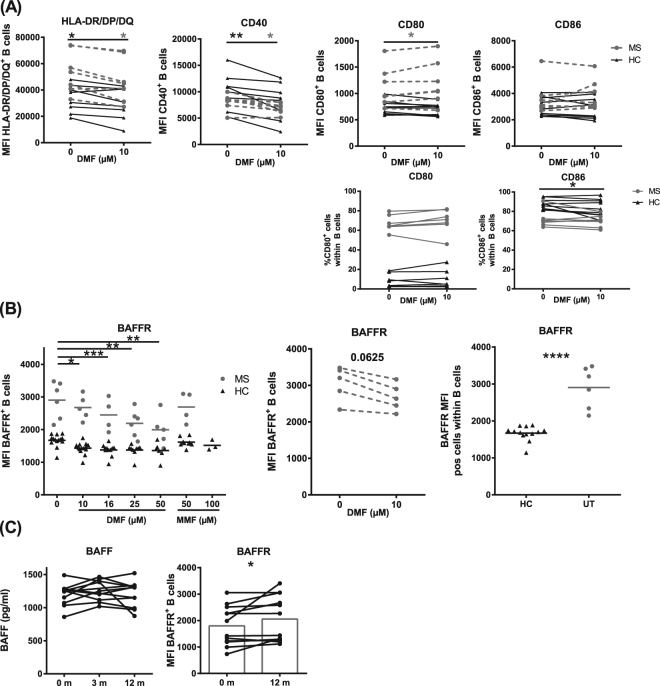
DMF downregulates the expression of antigen-presentation molecules, costimulatory molecule CD40 and survival marker BAFFR on B cells. (**A**) Purified B cells of HC and untreated RRMS patients were treated *in vitro* with 10 µM DMF or were left untreated. Expression (MFI) of HLA-DR/DP/DQ (HC: n = 8, MS: n = 7), CD40 (HC: n = 8, MS: n = 7), CD80 (HC: n = 9, MS: n = 7) and CD86 (HC: n = 9, MS: n = 7) on B cells and % CD80^+^ (HC: n = 8, MS: n = 7) and % CD86^+^ (HC: n = 9 MS: n = 7) B cells is depicted for HC and MS patients. (**B**) Purified B cells of HC and untreated RRMS patients were treated *in vitro* with the following concentrations of DMF: 10 µM (HC: n = 14, MS: n = 5), 16 µM (HC: n = 11, MS: n = 4), 25 µM (HC: n = 9, MS: n = 6) and 50 µM (HC: n = 7, MS: n = 5), or with MMF: 50 µM (HC: n = 9, MS: n = 4) or were left untreated (HC: n = 14 and MS: n = 5). (**C**) Plasma BAFF levels of RRMS patients at baseline (n = 11), 3 m of DMF treatment (n = 9) and 12 m of DMF treatment (n = 11) and expression (MFI) of BAFFR on B cells of RRMS patients at baseline and 12 m of DMF treatment (n = 12). A Kruskal-Wallis one-way ANOVA was used to compare the different groups and a Wilcoxon matched-pairs signed rank test was used to determine p values between two groups. ^*^p < 0.05, ^**^p < 0.01, ^***^p < 0.001, ^****^p < 0.0001. HLA-DR/DP/DQ = human leukocyte antigen, HC = healthy control, MS = multiple sclerosis, DMF = dimethyl fumarate, MFI = mean fluorescence intensity, BAFF(R) = B cell activating factor (receptor), pos = positive.

We previously demonstrated the potential of MS B cells to induce autoreactive T cell responses via antigen presentation and increased costimulatory molecule expression on MS B cells^4^. As immunomodulatory treatment abrogated these effects^4^, we investigated whether *in vitro* treatment with DMF directly decreased the expression of antigen presentation and costimulatory molecules (Table 1). DMF, but not MMF, directly decreased B cell expression of HLA-DR/DP/DQ and CD40 in HC (p = 0.0234 and p = 0.0078, respectively) and untreated MS patients (p = 0.0313 and p = 0.0313, respectively, Fig. 7A, Supplementary Fig. 5). In contrast, DMF increased CD80 expression on B cells of untreated MS patients (p = 0.0469), while no effect was detected in HC or on CD86 expression. However, DMF had no effect on the percentage of CD80^+^ B cells while it decreased the percentage of CD86^+^ B cells in HC.

B cell survival was further investigated via BAFFR expression. DMF, but not MMF, directly decreased BAFFR expression on B cells *in vitro*, already at 10 µM (HC: p < 0.05; MS: p = 0.0625) with stronger effects using higher DMF concentrations (p < 0.01, Fig. 7B). An increased baseline BAFFR expression level was observed on MS B cells compared to HC (p < 0.0001). In our follow-up study, an increased BAFFR expression was detected after 12 m of DMF treatment and no effect was measured on BAFF plasma levels (Fig. 7C). This suggests that DMF treatment has a direct effect on B cell survival by decreasing BAFFR expression and an indirect effect *ex vivo* due to a shift in B cell subtypes.

## Discussion

In 2013, DMF (Tecfidera^®^), was licensed as an oral first-line treatment for RRMS^15,16^. However, its mechanism of action is still not fully elucidated. Here, we used 3 study designs and demonstrated that DMF treatment induced a persistent change of both the innate and adaptive immune system of MS patients. Furthermore, DMF directly induced concentration-dependent apoptosis and reduced expression of functional markers of B cells.

Next to the immunological findings, clinical parameters were analyzed in the 12 m follow-up study. DMF treatment improved cognitive function (PASAT) and showed a trend towards improved EDSS of MS patients. Cognitive decline is a serious complication in MS that is characterized by deficits in (non)verbal memory, information processing speed and sustained attention^22^. However, validation in a larger cohort of DMF-treated MS patients is needed.

We further showed that DMF treatment selectively targets or has a stronger effect on particular immune cell subtypes, since it decreased total leukocyte and lymphocyte numbers without affecting monocytes, neutrophils and anti-inflammatory subtypes such as CD56^+^ NK cells and transitional B cells. Absolute numbers in this study are comparable to others^21,23–25^. The B cell subtype numbers are in line with Li *et al*. where DMF preferentially decreased mature B cells without affecting transitional B cells after at least 3 m of treatment^20^. However, our study reinforced this by showing a persistent change after 12 m. We further confirmed the preferential loss of CD8^+^ T cells (58%) versus CD4^+^ T cells (41%) and additionally indicated that this loss was persistent after 12 m of treatment. However, in our study none of the DMF-treated MS patients showed a decline of CD4^+^ T cells below the lower limit of normal (LLN, 400/µl) and only 14% of the patients showed a decline of CD8^+^ T cells below the LLN (200/µl), which is in contrast with other studies^24,25^. This could be due to the difference in patient numbers and inclusion criteria. After 3 m of DMF treatment, no significant changes were observed in the number of innate and adaptive immune cell subtypes, which is in agreement with Spencer *et al*.^25^.

As the absolute numbers of almost all immune cell subtypes decreased, it was important to consider their percentages to elucidate which subtypes were preferentially affected by DMF treatment. We showed that innate immune cell percentages (monocytes and NK cells) increased, implying that DMF prevails normal innate immune function. The relative increase in monocytes could be the result of the decreased absolute lymphocyte number, as the absolute monocyte number did not change. CD56^+^ NK cells were increased after DMF treatment, while CD56^dim^NK cells increased to a lesser extent. Medina *et al*. demonstrated an increase of CD56^+^ NK cells after 6 m of DMF treatment, but we reinforced this result by showing its persistence after 12 m of DMF treatment^26^. Furthermore, Chaves *et al*. and Medina *et al*. did not find a change in total NK cell percentages which indicates that it is important to investigate NK cell subtypes since CD56^+^ and CD56^dim^ NK cells were described to have different functions^26,27^. CD56^+^ NK cells were first considered regulatory NK cells by Cooper *et al*. due to a reduced cytotoxicity and an increased production of cytokines compared to cytotoxic CD56^dim^ NK cells^28^. Further, NK cells have become increasingly important as players in MS pathogenesis, as the expansion of CD56^+^ NK cells was previously correlated with the efficacy of daclizumab in MS patients^29^. Regulatory NK cells cytolyze activated T cells in a comparable way as cytotoxic NK cells, whereas they have no cytotoxicity towards resting T cells^29^. Since regulatory NK cells cytolyze activated T cells and their expansion is linked with the disease activity of MS patients, regulatory NK cells are clearly important in MS. As with daclizumab, the increase in regulatory NK cells is one of the beneficial mechanisms of DMF. Other mechanisms of DMF, including T cell apoptosis and inhibition of dendritic cell maturation, might indirectly be a consequence of this finding^21,30^.

Within the adaptive immune system, DMF treatment reduced percentages of pro-inflammatory immune cell subtypes such as (effector) memory (CD4^+^ and CD8^+^) T cells and (non) class-switched memory and DN B cells in the follow-up study. Furthermore, DMF treatment increased naive T and B cells and transitional B cells. This switch from a pro-inflammatory profile into an anti-inflammatory profile is in line with other studies^19,26,31,32^. Since the same results are obtained in different study populations of different countries (Spain, Germany, USA and Belgium) it appears that DMF has a rather stable immunological effect, although it has many mechanisms of action. In addition, our cross-sectional study emphasizes the long-lasting effect of DMF.

Medina *et al*. and Smith *et al*. reported a reduction of memory B cells and the latter also reported an increase in naive B cells at 6 m of treatment^26,33^. Our study additionally indicated a persistent effect of DMF after 12 m treatment on naive and memory B cells. In contrast to others, who reported no effect of DMF on transitional and Breg cells after 6 m of treatment^26,33^, we detected an increase in transitional B cells. This could indicate that the effect of DMF on transitional B cells is only detectable after a longer treatment period, which was confirmed by Lundy *et al*., who showed increased Breg cells in 4 of the 8 MS patients after 12 m of DMF treatment compared to 4–6 m of DMF treatment^34^. Furthermore, we can speculate that transitional and naive B cells are more resistant to DMF treatment than memory B cells, although we demonstrated that DMF directly induced B cell apoptosis without a preferential effect on memory or naive B cells. Indirect effects, such as the interplay between immune cells or the output of new B cells from the bone marrow, could be responsible for the findings of the follow-up study. Transitional B cells are enriched for Breg which could have beneficial effects in MS^3^. Furthermore, results from *in vitro* treatment of B cells with DMF induced increased Breg percentages, although not significantly. In addition, MMF induced a trend towards a decreased percentage of TNF-α^+^ B cells. Thus, the increased Breg percentage^34^ and decreased percentage of TNF-α^+^ B cells^20,33^ that were previously described after DMF treatment could be regulated in a direct manner. As Breg are known to suppress Th1 and Th17 differentiation while inducing Treg generation^35^, the decreased IFN-γ^+^ and IL-17^+^ CD4^+^ T cells after DMF treatment in the follow-up study could be an indirect consequence of an increase in Breg.

When considering CD4^+^ T cells expressing the anti-inflammatory cytokines IL-4 and IL-10, we could not find a difference after treatment with DMF. One explanation could be the great patient diversity in IL-4^+^ and IL-10^+^ CD4^+^ T cells. Because of this variety a population-wide conclusion about the effect of DMF on IL-4^+^ and IL-10^+^ CD4^+^ T cells cannot be made. Other studies also did not detect a difference in the percentage of IL-4^+^ or IL-10^+^ CD4^+^ T cells^19,26,36^. In contrast, Wu *et al*. detected an increase in IL-4^+^ T cells, although the percentage of IL-4^+^ T cells was much lower than detected in other studies^32^.

One of the limitations of our study is that we did not include memory CD4^+^ cytokine expressing cells. Since we observed a decrease in pro-inflammatory cytokine expressing CD4^+^ cells it is still possible that this reduction is due to the reduction of memory CD4^+^ cells observed in the follow-up study. Furthermore, it would have been interesting to assess CD8^+^ cytokine expressing T cells.

The direct effect of DMF on B cells was studied in more detail. Gillard *et al*. demonstrated that DMF and MMF are active between 5.5 and 50 µM and 50 and 150 µM, respectively. Therefore, DMF was used at different concentrations between 10 µM and 50 µM, while 50 µM of MMF was used^37^. DMF induced concentration-dependent B cell apoptosis in HC and untreated RRMS patients starting from a concentration of 25 µM DMF. As MMF did not have an effect on B cell apoptosis, DMF is responsible for the full effect on the reduction of B cell numbers. Although DMF could not be detected in serum of HC, a degree of systemic penetrance of DMF has been reported with DMF-glutathione conjugates found in urine of DMF-treated psoriasis patients^38^. Furthermore, Litjens *et al*. demonstrated that DMF could be taken up by lymphocytes and monocytes^39^.

Due to the fact that the exact concentration of DMF in the circulation or in immune cells of RRMS patients is not known and cannot be determined exactly, it is important to investigate the direct effects of DMF on B cells in low concentrations *in vitro*. Since Blewett *et al*. demonstrated that DMF directly interacts with cysteine residues of T cells in concentrations between 10 and 50 µM and that this is mechanistically relevant to the immunosuppressive activity of the drug and since we have demonstrated that no apoptosis is induced in concentrations below 25 µM, we examined whether DMF also exerted a direct effect on functional markers of B cells at a concentration of 10 µM^40^. In this study, we demonstrated that DMF directly decreased B cell expression of antigen presentation HLA-DR/DP/DQ and costimulatory molecule CD40. This could lead to reduced T cell activation, since B cells are critical antigen presenting cells in MS that can stimulate T cell proliferation to foreign or neuro-antigens^4^. Previous reports demonstrated increased CD40 ligand on MS T cells and hyper-responsiveness of MS memory B cells to CD40 stimulation^41,42^. Therefore, we suggest that the decreased B cell CD40 expression induced by DMF will result in disrupted B cell activation and thus B cell mediated pathology. Furthermore, these results could explain the decrease in memory T cells and reduced T cell proliferation and activation following DMF treatment^23,27,31,32,43^.

DMF directly reduced B cell expression of the survival marker BAFFR. However, BAFF serum levels were unchanged and B cell BAFFR expression was increased in the follow-up study. The latter effect could be due to increased transitional and naive B cells after DMF treatment. BAFF has been described to play a dual role in MS pathology including both pathological effects^44^ and positive effects on treatment outcome and detrimental effects of BAFF antagonists^45^. Interestingly, BAFFR expression was increased on B cells of untreated MS patients compared to HC. An increased BAFFR mRNA level was previously demonstrated in MS patients^46^. BAFF is important for maintaining antibody-producing plasma cells and the direct effect of DMF on BAFFR expression could be beneficial for MS pathogenesis.

In conclusion, this study shows that DMF treatment persistently changes the immune balance of MS patients and illustrates the multifactorial working mechanism of DMF. The effects of DMF elucidated here could help explain its therapeutic efficacy in MS.

## Methods

### Study subjects

RRMS patients and healthy controls (HC) were recruited at the Zuyderland Medical Center (Sittard, The Netherlands), Rehabilitation & MS-Center (Overpelt, Belgium) and Biomedical Research Institute (Diepenbeek, Belgium). All experimental protocols and methods were conducted in accordance with institutional guidelines and regulations and was approved by the institutes’ ethics committees (METC14-T-96, 15.143/neuro15.12) and all study subjects gave written informed consent. For the follow-up study, peripheral blood was collected before, after 3 m and 12 m of DMF treatment. Clinical outcome measures including the Expanded Disability Status Scale (EDSS) and the Fatigue Scale for Motor and Cognitive Functions (FSMC) were analyzed. Furthermore, the timed 25-foot walk (T25FW), the Paced Auditory Serial Addition Test (PASAT-3″) and the Nine Hole Peg test (NHPT) were analyzed according to the Multiple Sclerosis Functional Composite (MSFC). All samples were stored in the University Biobank Limburg. Further clinical details and numbers of MS patients and HC are provided in Table 1. In all analyses, HC were matched to MS patients as closely as possible with regard to age and sex.

### Cell isolation

Peripheral blood mononuclear cells (PBMC) were isolated from whole blood by Ficoll density gradient centrifugation (Lympholyte, Cedarlane Laboratories, Uden, The Netherlands). PBMC of MS patients and HC were cryopreserved and thawed before use, unless stated otherwise. PBMC collected at different time points during the follow-up study were simultaneously thawed to exclude inter-assay variation. PBMC were cryopreserved in 10% dimethyl sulfoxide (DMSO, Sigma Aldrich, Overijse, Belgium) and fetal bovine serum (FBS, Life Technologies, Gent, Belgium) using a slow temperature-lowering method (Coolcell®, VWR, Haasrode, Belgium). After 24 h, cryovials were transferred to liquid N_2_ until analysis. PBMC were thawed by bringing the temperature of the cryovials to 0 °C in a water bath (37 °C). Hereafter, cold thawing medium consisting of 20% FBS in RPMI 1640 (Lonza, Basel, Switzerland) was added and PBMC were centrifuged at 4 °C. PBMC were suspended in thawing medium in a concentration of 10 × 10^6^ cells/1000 µl containing DNAse (1/100) and incubated at 37 °C for 10 minutes. Subsequently, cells were washed twice. Median cell recovery was 76 ± 18%. B cells were purified from freshly isolated PBMC by negative magnetic selection (STEMCELL Technologies SARL, Grenoble, France). Purity of B cells was routinely ≥98.4%.

### Flow cytometry

In the follow-up study, PBMC were stained with anti-human CD14 PerCP, CD16 FITC, CD56 PE-Cy7 (BD Biosciences, Erembodegem, Belgium) and CD3 APC-Cy7 (BioLegend, Antwerp, Belgium). B cell subtypes were analyzed using anti-human CD19 PE-Cy7, immunoglobulin (Ig)M PerCP-Cy5.5 (BioLegend), CD27 APC, IgD PE-CF594, IgG FITC (BD Biosciences), CD38 APC-ef780 (eBioscience, San Diego, USA) and IgA PE (Miltenyi Biotec, Leiden, The Netherlands). T cell subtypes were analyzed with anti-human CD4 APC (BioLegend), CD45RA APC-ef780, CD127 PE, CD25 PE-Cy7 (eBioscience), CD45RO PE-CF594, CD8 FITC and CCR7 PerCP-Cy5.5 (BD Biosciences). Expression of B-cell activating factor receptor (BAFFR) on B cells was assessed using CD19 PE-Cy7, CD268 APC-Cy7 (BAFFR) (BioLegend).

For intracellular T cell cytokine analysis, PBMC were stimulated with phorbol 12-myristate 13-acetate (PMA, 50 ng/ml) and calcium ionomycin (1 µg/ml, Sigma-Aldrich, Overijse Belgium) in the presence of Golgistop (BD Biosciences) for 4 h. PBMC were fixed and permeabilized using Cytofix/Cytoperm (BD Biosciences) and stained with anti-human IL-10 PE-Cy7, GM-CSF APC, CD4 PE-Cy5 (BioLegend), IL-4 PE, IL-17 FITC (eBioscience) and IFN-γ PE-CF594 (BD Biosciences). Freshly isolated PBMC were analyzed in the cross-sectional study using anti-human CD4 APC, CD8 PE, CD45RO (BD Biosciences). Apoptosis and BAFFR expression of *in vitro* cultured B cells was measured using anti-human CD19 PE-Cy7, CD268 APC-Cy7 (BioLegend), CD27 APC, IgG PE, IgD PE-CF594 (BD Biosciences), annexin V FITC and 7-AAD (eBioscience). Expression of costimulatory and antigen presentation molecules was assessed using anti-human CD19 PerCP-Cy5.5, CD27 APC, CD80 PE, CD86 PE-CF594, HLA-DR/DP/DQ FITC (BD Biosciences) and CD40 PE-Cy7 (eBioscience). When analyzing frequency of Breg and pro-inflammatory B cells, B cells were restimulated during the last 4 h with PMA (50 ng/ml) and calcium ionomycin (1 µg/ml, both Sigma-Aldrich) in the presence of GolgiStop (BD Biosciences). Fixation and permeabilization were done using Cytofix/Cytoperm (BD Biosciences). The antibodies used were anti-human CD19 BV421, CD24 PE-Cy7, IL-10 Brilliant Violet 711 (all from BD Biosciences), CD38 APC-ef780, Fixable Viability Dye-eFluor506 (all from eBioscience) and TNF-α PerCP-Cy5.5 (BioLegend). All flow cytometry was performed on a FACSAria II or LSRFortessa flow cytometer using FACSDiva software (BD Biosciences). Absolute numbers of leukocytes, lymphocytes, monocytes and neutrophils were obtained from standard clinical lab tests for 7 DMF-treated MS patients. Absolute numbers of lymphocyte subtype populations were calculated as follows: absolute lymphocyte number (10^9^/l) × percentage of lymphocyte subtype population × 10.

### *In vitro* B cell cultures

B cells were cultured in 96-well round-bottom plates (Greiner Bio-One B.V., Alphen aan den Rijn, The Netherlands) at 3 × 10^5^ cells in RPMI 1640 (Lonza, Basel, Switzerland) with 10% fetal bovine serum (FBS, Life Technologies, Gent, Belgium), 50 U/ml penicillin, 50 mg/ml streptomycin (Invitrogen, Carlsbad, CA), 0.1 mM nonessential amino acids and 1 mM sodium pyruvate (Sigma-Aldrich). For analysis of B cell apoptosis, survival marker and expression of costimulatory and antigen presentation molecules, B cells were treated during 24 h with DMF (10 µM–50 µM), MMF (50 µM) or were left untreated (DMSO). After 1 h, B cells were stimulated with 2 µg/ml CpG2006 (ODN2006, Invivogen, Toulouse, France) for the following 23 h. When measuring the frequency of Breg and pro-inflammatory B cells, purified B cells were treated during 48 h with DMF (10 µM), MMF (50 µM) or were left untreated in the presence of 1 µg/ml soluble CD40 ligand together with 2 µg/ml CpG2006.

### ELISA

BAFF plasma levels were quantified before and after 12 m of DMF treatment using a commercial ELISA kit according to the manufacturer’s protocol (R&D systems, Abingdon, United Kingdom).

### Statistics

Statistical analyses were performed using Graphpad Prism 6. Wilcoxon matched-pairs signed rank test was used for comparison of variables at baseline and follow-up and for comparing cells with or without DMF treatment (*in vitro*). When comparing multiple groups, one-way ANOVA (Kruskal-Wallis) was used. A p value less than 0.05 was considered statistically significant.

## Electronic supplementary material

Supplementary information
