# Supplementary material for: Dimethyl fumarate induces a persistent change in the composition of the innate and adaptive immune system in multiple sclerosis patients
Source: Sci Rep. 2018 May 29;8:8194. doi: 10.1038/s41598-018-26519-w (PMC5974280; doi:10.1038/s41598-018-26519-w)
Supplement: Supplementary file 1 — Supplementary information [file 41598_2018_26519_MOESM1_ESM.pdf]

Supplementary Material

Dimethyl fumarate induces a persistent change in the composition of the innate and adaptive immune system in multiple sclerosis patients

Gwendoline Montes Diaz, Judith Fraussen, Bart Van Wijmeersch, Raymond Hupperts and Veerle Somers

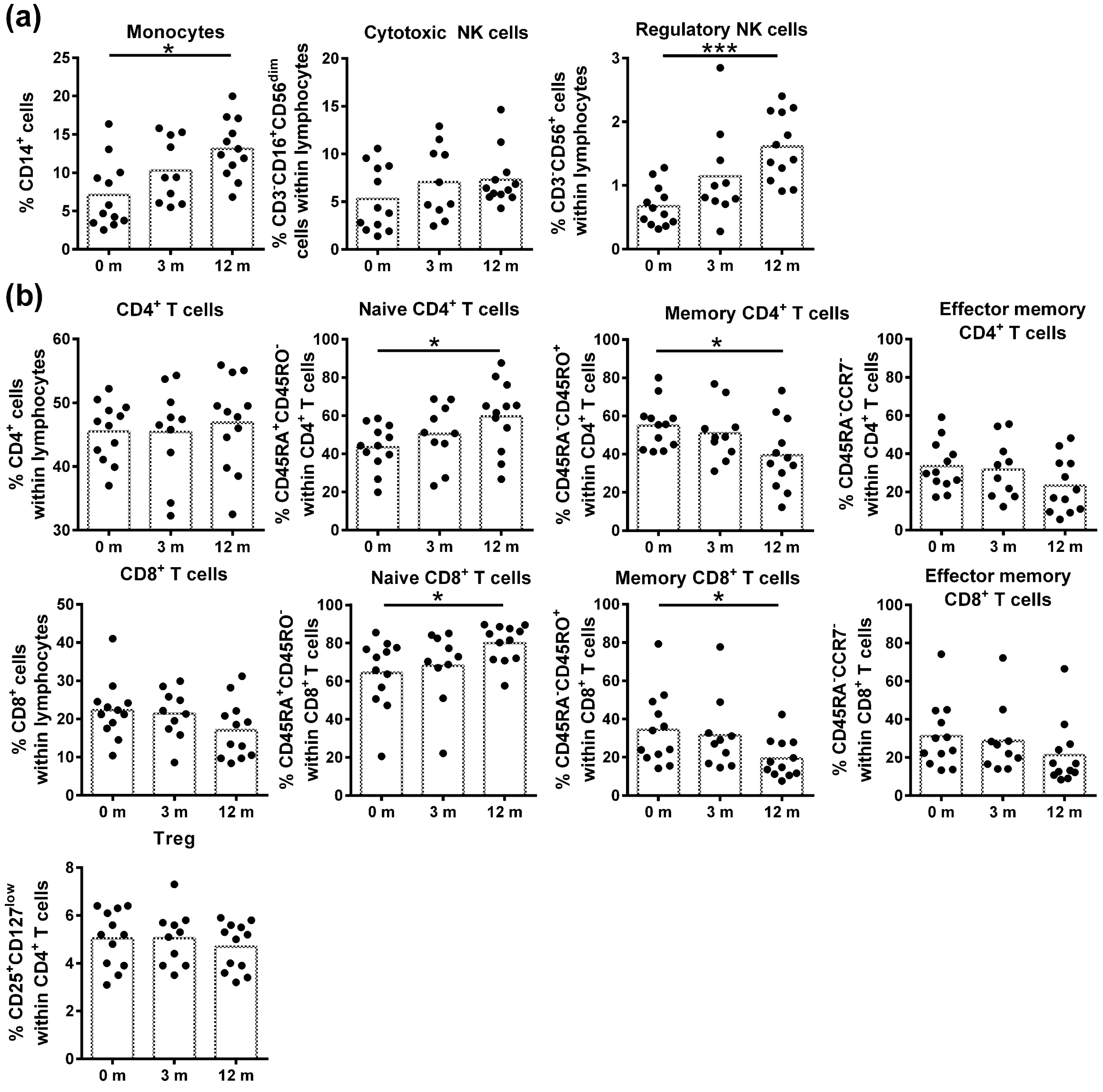

(c)

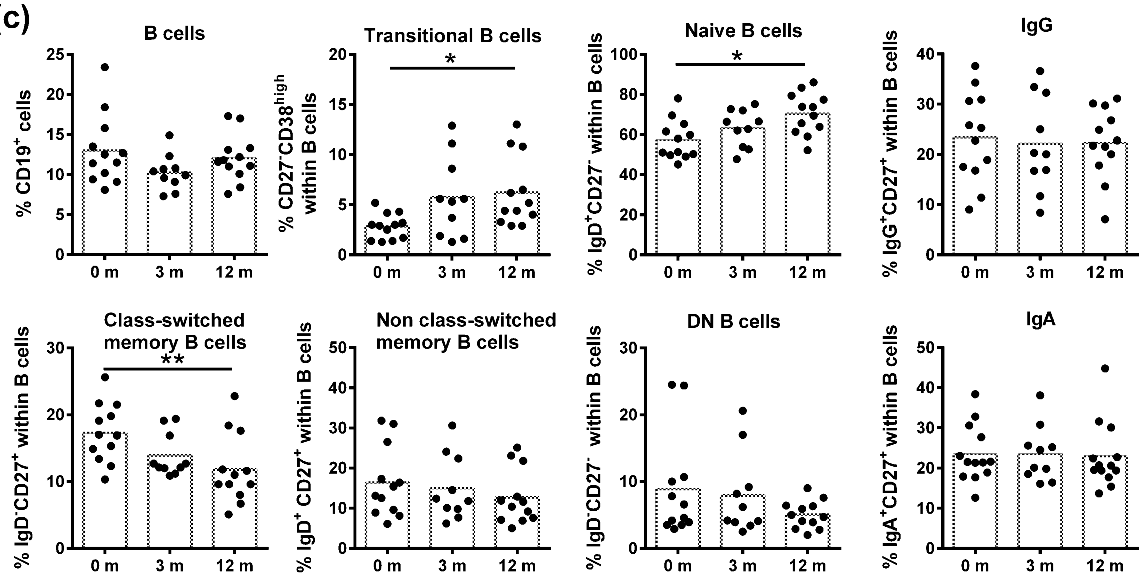

Supplementary Figure 1. DMF treatment induced redistribution of innate and adaptive immune cell subtypes after 12 m of treatment in RRMS patients, while no significant changes were detected at 3 m of DMF treatment. (a) Frequency of monocytes, NK cells, **(b)** T cell subtypes and **(c)** B cell subtypes at baseline (n = 12), after 3 m (n = 10) and after 12 m (n = 12) of DMF treatment in RRMS patients. Each dot represents an individual patient and average values are depicted as histograms. A Kruskal-Wallis one-way ANOVA test was used to determine p values. * $p < 0.05$, ** $p < 0.01$, *** $p < 0.001$. NK = natural killer, Treg = regulatory T cells, DN = double negative, m = months.

(a)

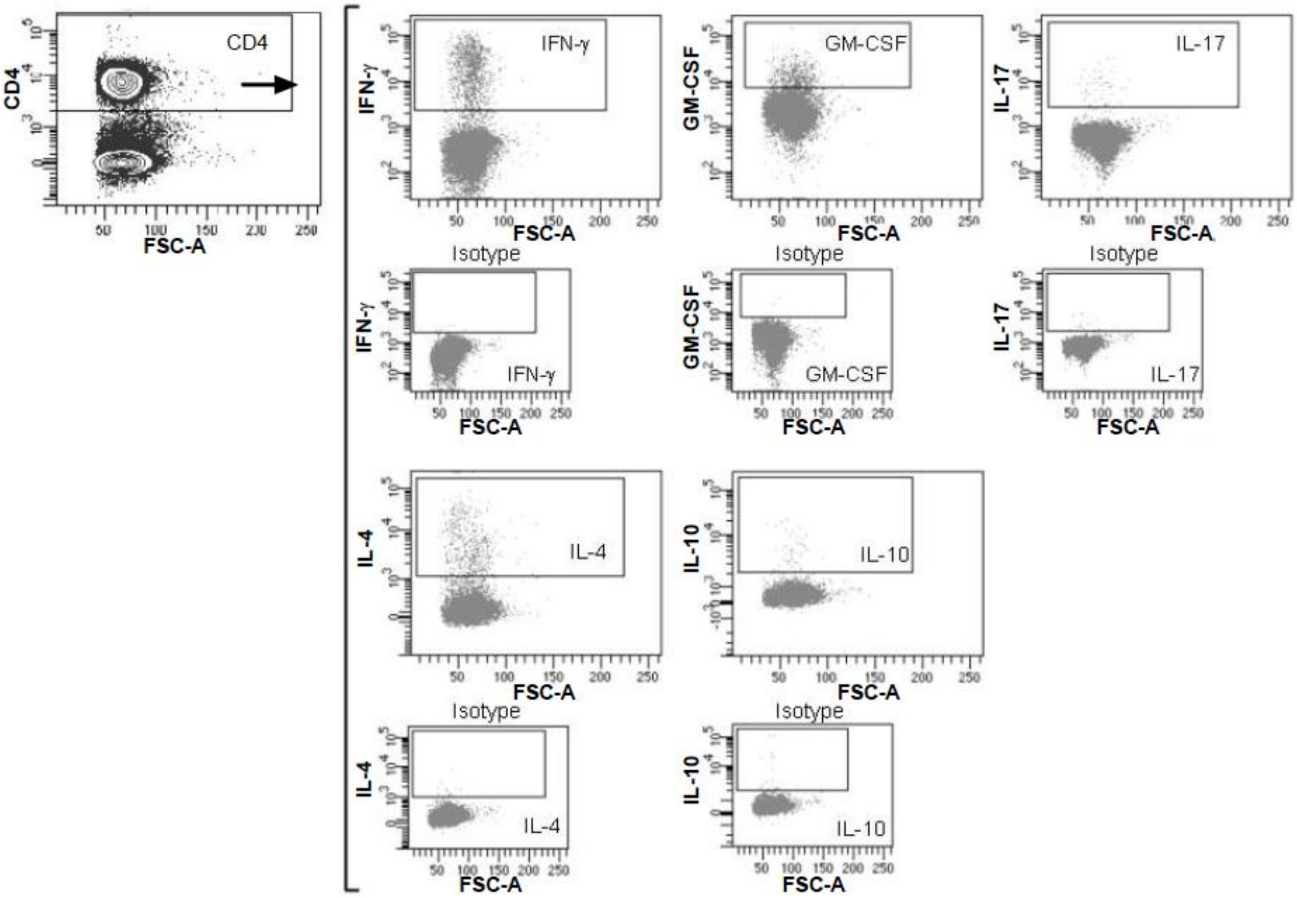
CD4
FSC-A
IFN-γ
GM-CSF
IL-17
Isotype
IL-4
IL-10

(b)

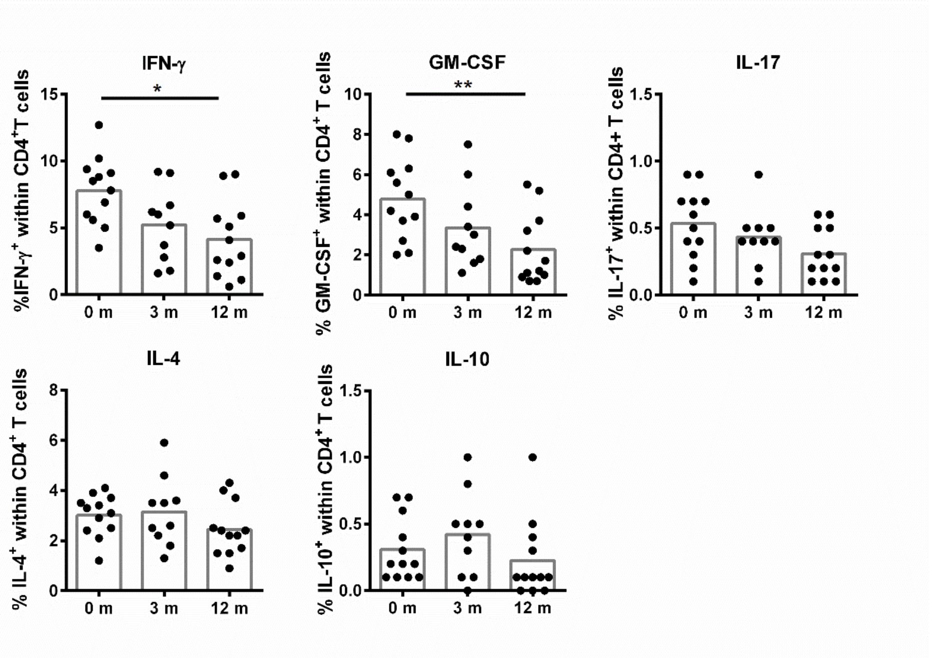
IFN-γ
GM-CSF
IL-17
IL-4
IL-10
%IFN-γ+ within CD4+T cells
% GM-CSF+ within CD4+ T cells
% IL-17+ within CD4+ T cells
% IL-4+ within CD4+ T cells
% IL-10+ within CD4+ T cells
0 m
3 m
12 m
*
**

Supplementary Figure 2. DMF treatment reduced frequency of T cells expressing pro-inflammatory cytokines after 12 m, but not after 3 m of treatment. (a) A representative analysis of cytokine-expressing T cell subtypes is shown. $CD4^+$ T cells were gated from lymphocytes. IFN-γ^+, GM-CSF^+, IL-17^+, IL-10^+ and IL-4^+ cells were gated from the $CD4^+$ cell population based on isotype controls **(b)** Frequency of $CD4^+$ T cells expressing pro-inflammatory cytokines (IFN-γ, GM-CSF, IL-17) and anti-inflammatory cytokines (IL-4, IL-10) in RRMS patients at baseline (n = 12), after 3 m (n = 10) and after 12 m (n = 12) of DMF treatment. Each dot represents an individual patient and average values are depicted as histograms. A Kruskal-Wallis one-way ANOVA test was used to determine p values. * $p < 0.05$, ** $p < 0.01$. IFN-γ = interferon-γ, GM-CSF = granulocyte macrophage colony-stimulating factor, IL-17 = interleukin-17, IL-4 = interleukin-4, IL-10 = interleukin-10, m = months.

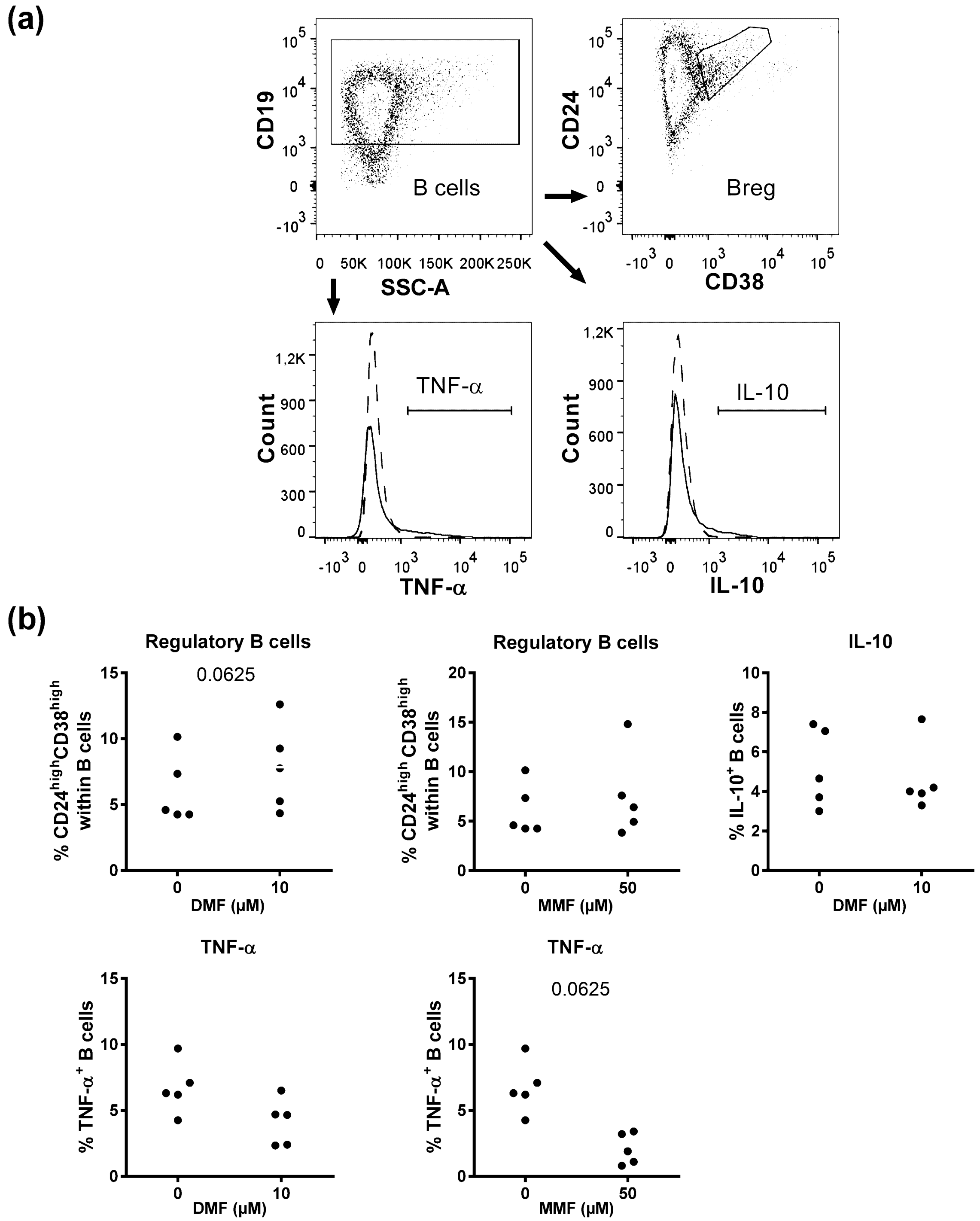

Supplementary Figure 3. A trend towards an increase in Breg is induced by DMF while MMF tends to decrease TNF-α⁺ B cells in a direct manner. Purified B cells of untreated RRMS patients were treated *in vitro* with 10 µM DMF, 50 µM MMF or were left untreated (n = 5). (a) A representative analysis of Breg and B cell cytokines is shown. Breg ($CD24^{high}CD38^{high}$), IL-10^+ and TNF-α^+ cells were gated from the B cell population based on unstimulated controls (dashed lines). (b) Frequencies of Breg ($CD24^{high}CD38^{high}$), IL-10^+ B cells and TNF-α^+ B cells are depicted. Wilcoxon matched-pairs signed rank test was used to determine p values. DMF = dimethyl fumarate, MMF = monomethyl fumarate, TNF = tumor necrosis factor.

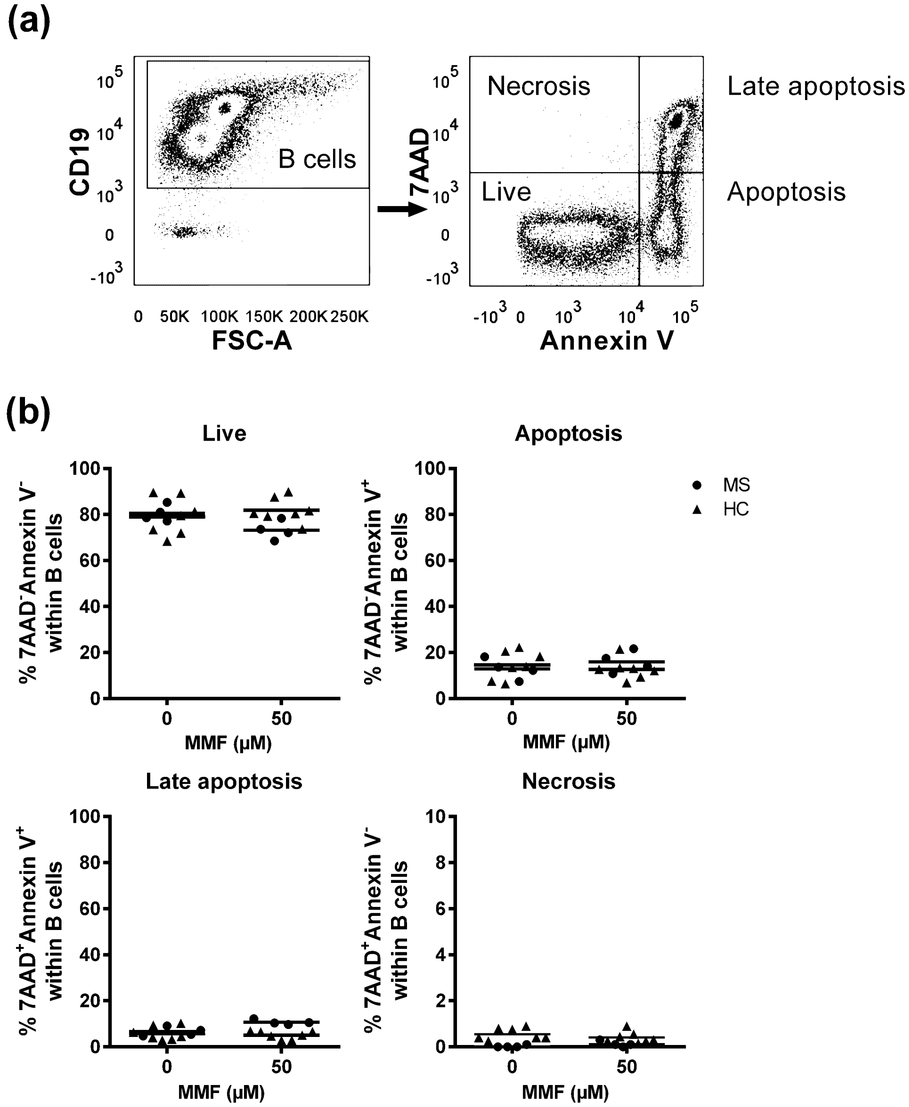

Supplementary Figure 4. MMF has no effect on apoptosis of B cells. Purified B cells of HC and untreated RRMS patients were treated *in vitro* with 50 µM MMF or left untreated (HC: n = 7, MS: n = 4). (a) A representative analysis of apoptosis of B cells is shown. Live (Annexin V^-7-AAD^-), apoptotic (Annexin V^+7-AAD^-), late apoptotic (Annexin V^+7-AAD^+) and necrotic (Annexin V^-7-AAD^+) cells were gated from the B cell population. (b) Frequency of live B cells (Annexin V^-7-AAD^-), apoptotic B cells (Annexin V^+7-AAD^-), late apoptotic B cells (Annexin V^+7-AAD^+) and necrotic B cells (Annexin V^-7-AAD^+) of HC and MS patients. Wilcoxon matched-pairs signed rank test was used. HC = healthy control, MS = multiple sclerosis, MMF = monomethyl fumarate.

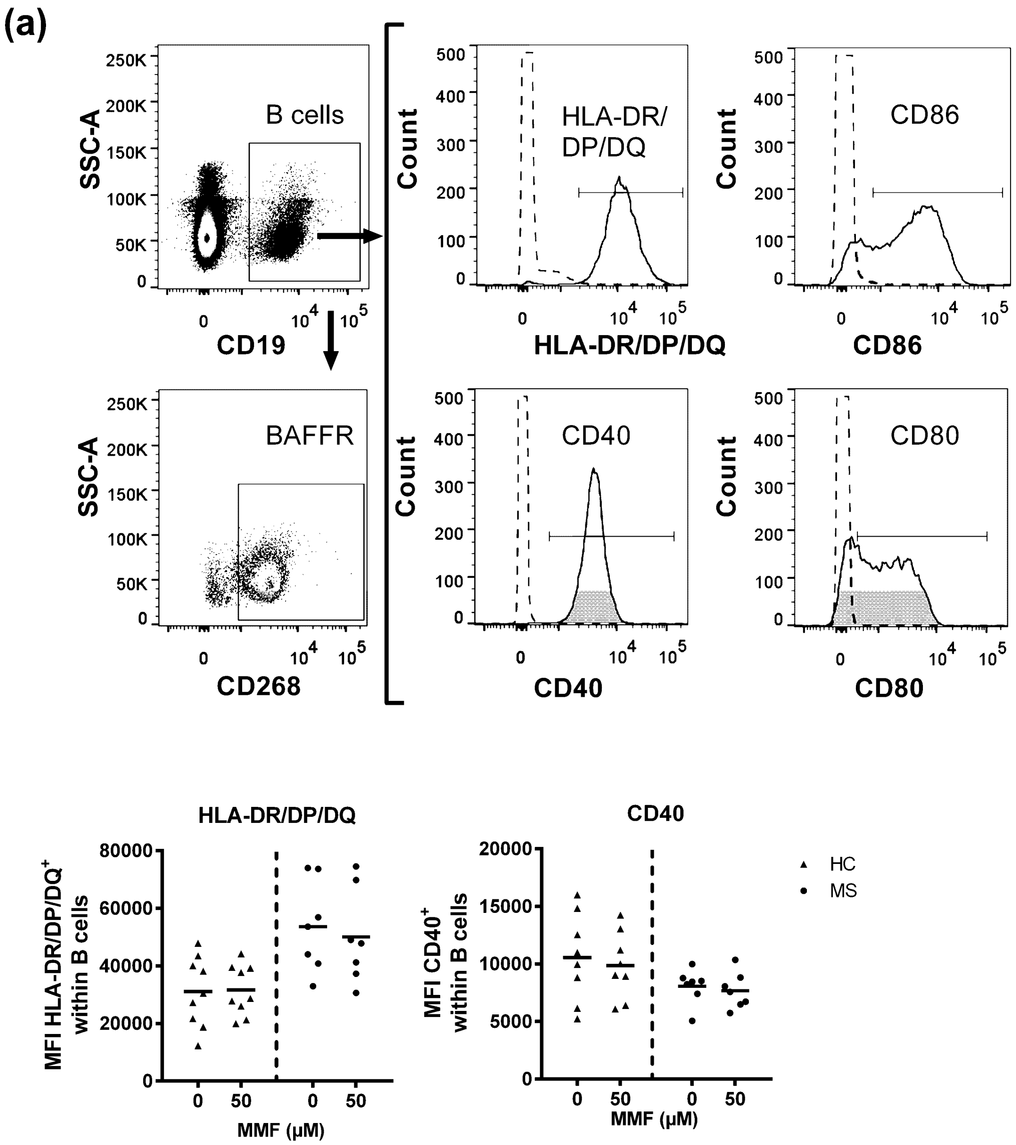

Supplementary Figure 5. MMF has no effect on the expression of antigen presentation molecules and costimulatory molecule CD40 of B cells. Purified B cells of HC and untreated RRMS patients were treated *in vitro* with 50 µM MMF or were left untreated. (a) A representative analysis of functional molecules expressed by B cells is shown. Survival marker BAFFR, antigen presentation molecule HLA-DR/DP/DQ and costimulation molecules CD40, CD80 and CD86 were gated from the B cell population based on isotypes controls (dashed lines). (b) Expression (MFI) of HLA-DR/DP/DQ (HC: n = 9, MS: n = 7), CD40 (HC: n = 8, MS: n = 7) on B cells is depicted for HC and MS patients. A Wilcoxon matched-pairs signed rank test was used to determine p values between two groups. HLA-DR/DP/DQ = human leukocyte antigen, HC = healthy control, MS = multiple sclerosis, MMF = monomethyl fumarate, MFI = mean fluorescence intensity.
